# U-Omp19 from *Brucella abortus* increases dmLT immunogenicity and improves protection against *Escherichia coli* heat-labile toxin (LT) oral challenge

**DOI:** 10.1016/j.vaccine.2020.05.039

**Published:** 2020-07-06

**Authors:** Lorena M. Coria, Franco L. Martinez, Laura A. Bruno, Karina A. Pasquevich, Juliana Cassataro

**Affiliations:** Instituto de Investigaciones Biotecnológicas, Universidad Nacional de San Martín (UNSAM), Consejo Nacional de Investigaciones Científicas y Técnicas (CONICET), Buenos Aires, Argentina

**Keywords:** Adjuvant, Oral vaccine, ETEC vaccine, U-Omp19, dmLT, Protease inhibitor

## Abstract

Acute diarrhea disease caused by bacterial infections is a major global health problem. Enterotoxigenic *Escherichia coli* (ETEC) is one of the top causes of diarrhea-associated morbidity and mortality in young children and travelers to low-income countries. There are currently no licensed vaccines for ETEC. Induction of immunity at the site of entry of the bacteria is key to prevent infection. Current approaches to ETEC vaccines include a less toxic mutant form of *E. coli* heat-labile toxin (double-mutant heat-labile enterotoxin -dmLT-) with both antigenic and immunostimulatory properties. U-Omp19 is a protease inhibitor from *Brucella* spp. with immunostimulatory properties that has been used as oral adjuvant. In this work, we use U-Omp19 as adjuvant in an oral vaccine formulation against ETEC containing dmLT in outbred and inbred mice. To evaluate antigen dose sparing by U-Omp19 three different immunization protocols with three different doses of dmLT were evaluated. We demonstrated that U-Omp19 co-delivery increases anti-LT IgA in feces using a mid-dose of dmLT following a prime-boost protocol (after one or two boosts). Oral immunization with U-Omp19 induced protection against LT challenge when co-formulated with dmLT in CD-1 and BALB/c mice. Indeed, there was a significant increase in anti-LT IgG and IgA avidity after a single oral administration of dmLT plus U-Omp19 in comparison with dmLT delivered alone. Interestingly, sera from dmLT plus U-Omp19 vaccinated mice significantly neutralize LT effect on intestine inflammation *in vivo* compared with sera from the group immunized with dmLT alone. These results demonstrate the adjuvant capacity of U-Omp19 to increase dmLT immunogenicity by the oral route and support its use in an oral subunit vaccine formulation against ETEC.

## Highlights

• Oral co-administration of dmLT with U-Omp19 increases dmLT immunogenicity.
• U-Omp19 oral co-delivery with dmLT induces anti-LT antibody responses.
• U-Omp19 co-administered with dmLT protects against oral challenge with LT.
• U-Omp19 can allow antigen dose sparing by oral route.
• U-Omp19 can be used as adjuvant in an oral vaccine formulation against ETEC.

## 1 Introduction

Acute enteric infections causing diarrhea and gastroenteritis constitute a global public health problem with high mortality and morbidity, particularly among children in low-income and lower middle-income countries. Diarrhea ranked ninth among causes of death for all ages, and fourth among infants, accounting for an estimated 499,000 deaths in children under 5 years old [1]. Enterotoxigenic *Escherichia coli* (ETEC) is among the top five pathogens that cause diarrheal mortality in children and it also causes significant burden across all ages [2].

ETEC causes a secretory diarrhea that can range in presentation from mild discomfort to a cholera-like illness. Transmission of ETEC person-to-person occurs via ingestion of faecally-contaminated food or water. In developed countries where sanitation standards are usually higher, ETEC infection is rare. However, it remains a leading cause of travelers’ diarrhea which occurs in people visiting or returning from ETEC-endemic regions [3], [4]. Epidemics of ETEC diarrhea have also occurred during natural disasters, such as floods where the quality of drinking water and sanitation were drastically affected [5].

This pathogen cause disease by colonization of the gut through colonization factors (CFs), most of which are fimbriae that promote the attachment of bacteria to host epithelial cells. They also produce and release enterotoxins (heat labile enterotoxin -LT- and/or a non-immunogenic polypeptide heat-stable enterotoxin -ST-) that disrupt fluid and electrolyte homeostasis in the small intestine, leading to fluid hypersecretion and watery diarrhea [6].

Conventional treatment of symptoms includes the use of oral rehydration salts (ORS) and, where appropriate and available, the use of antimicrobials. However, with the emergence of multi-drug resistant strains of ETEC, the need for vaccines against this pathogen is increased [7]. At present there is no vaccine specifically licensed to prevent ETEC disease. The oral killed whole-cell cholera vaccine, Dukoral, which is available for travelers in Canada and Europe, contains the recombinant cholera toxin subunit B, which is homologous with LT of ETEC and by extension provides partial protection against this bacterium. Unfortunately, most ETEC strains express or co-express ST [5], [8].

Many alternative vaccine candidates designed specifically to protect people against ETEC diarrhea are under clinical development. Potential vaccines can be divided into two groups: inactivated vaccines containing killed whole cells, purified CF antigens, or inactivated LT; and live attenuated vaccines containing genetically modified, nonpathogenic strains of ETEC or alternative carrier bacteria expressing the important ETEC antigens [9], [10]. Most vaccine formulations have been based on LT or CFs from ETEC since it has been reported that both antitoxin and antibacterial antibodies are important to confer protection [11], [12]. Vaccine candidates including ETEC adhesins have also demonstrated be protective [13].

Anti-LT antibodies are important to protect against ETEC diarrheal disease as has been evidenced in ETEC challenge studies in human adults and in infants naturally receiving breast milk containing anti-LT IgA. These results suggested that antibodies can provide immunity against toxigenic effect of LT and possibly avoid ETEC colonization [14], [15]. In the same way the drop of diarrheal illness after five years of age in endemic regions correlates with anti-LT antibody responses [16], [17], [18].

Heat-labile enterotoxin has been studied as a potential vaccine antigen (Ag) and adjuvant [19], [20] but its toxicity limits its use in humans. Less toxic derivate forms have been developed, the most relevant is attenuated double mutant heat-labile toxin LTR192G/L211A (dmLT) that has a reduced toxigenic effect that allows its use in humans [20], [21], [22]. dmLT has both antigenic and adjuvant properties and it has been proved to be safe in oral and sublingual studies, currently is being tested for intradermal delivery [23], [24], [25], [26]. The most advanced oral ETEC vaccine candidate (ETVAX) is a tetravalent, inactivated whole-cell ETEC vaccine containing dmLT, currently under phase 2 clinical trial [27]. Recently it has been published that dmLT can enhance the protective efficacy of an orally delivered live attenuated vaccine expressing CS/CFA antigens in humans [28].

At present, there are no clinical studies using oral subunit vaccine formulations against ETEC with dmLT as antigen. ETEC oral vaccines containing dmLT as adjuvant are whole cell vaccines [29], [30] and dmLT as adjuvant in subunit vaccines has been assessed only for parenteral vaccines. Previous studies reported that a single oral dmLT dose of up to 100 µg is well tolerated in human subjects. Although immune responses were better after a single dose of 50 µg in humans [31]. Development of effective vaccines administered by oral route are restricted by the highly acidic and degradative gastrointestinal ambient where Ags are denatured or degraded. However, this route should not be dismissed since effective mucosal immunogenicity can be achieved by an appropriate Ag formulation that can prevent vaccine degradation at the gut.

In our previous work we demonstrated that a bacterial protease inhibitor from *Brucella abortus* (U-Omp19) can be used as an adjuvant in oral vaccine formulations [32]. Co-administration of U-Omp19 with an Ag can (i) bypass the harsh environment of the gastrointestinal tract partially inhibiting stomach and gut proteases and consequently it increases the half-life of co-delivered Ags while (ii) induces the recruitment, activation and increase Ag half-life inside Ag presenting cells (APCs) [33]. Besides, it increases the amount of Ag bearing dendritic cells at inductive sites increasing Ag immunogenicity [34]. Likewise U-Omp19 improves protection against *Toxoplasma gondii* and *Salmonella Typhimurium* challenge when is co-administered with subunit Ags in different murine models [32], [33], [35], [36]. Of note we have shown by circular dichroism that U-Omp19 is pH- and temperature-resistant [33]. Recently we demonstrated that U-Omp19 inhibits protease activity from murine intestinal brush-border membranes and cysteine proteases from human intestinal epithelial cells (IECs) promoting co-administered Ag accumulation within lysosomal compartments of IECs. In addition, we have shown that co-administration of U-Omp19 facilitated the transcellular passage of Ag through epithelial cell monolayers *in vitro* and *in vivo* while did not affect epithelial cell barrier permeability. Finally, oral co-delivery of U-Omp19 in mice induced the increment of CD103^+^ CD11b^−^ CD8α^+^ dendritic cells subset at Peyer's patches. Ag oral administration with U-Omp19 increases the frequency of mucosal DCs bearing the co-delivered Ag [37].

In this work we will evaluate the capacity of U-Omp19 to increase immune responses against LT from ETEC and its ability to improve protection against LT challenge when is combined with dmLT in an oral vaccine formulation. We will also evaluate if U-Omp19 can allow dmLT dose sparing by the oral route.

## 2 Material and methods

### 2.1 Ethics statement

All experimental protocols were conducted in agreement with international ethical standards for animal experimentation (Helsinki Declaration and its amendments, Amsterdam Protocol of welfare, and animal protection and National Institutes of Health, USA, guidelines: Guide for the Care and Use of Laboratory Animals). The protocols used were approved by the Institutional Committee for the Care and Use of Experimentation Animals (CICUAE) from the University of San Martin (UNSAM) (Permit Number: 04-2016), Buenos Aires, Argentina.

### 2.2 Animals

Eight to twelve-week-old female BALB/c mice were obtained from the Animal Facility of Instituto de Investigaciones Biotecnológicas (IIB-UNSAM). CD-1 mice were purchased from Charles River (USA). Mice were housed in appropriate conventional animal care facilities and handled according to international guidelines required for animal experiments at IIB-UNSAM.

### 2.3 Immunogen and adjuvants

Attenuated double mutant heat-labile toxin LTR192G/L211A (dmLT) was provided by PATH (Seattle, US) and used as immunogen [31]. Recombinant U-Omp19 was expressed and purified as previously described [32]. LPS contamination from U-Omp19 was adsorbed with Sepharose-Polymyxin B (Sigma). Endotoxin determination was performed with Limulus amoebocyte chromogenic assay (LONZA). All U-Omp19 preparations used contained < 0.1 endotoxin units per mg of protein. Heat-labile enterotoxin (LT) was provided by John Clements (Tulane University, New Orleans, US).

### 2.4 Immunizations

Inbred female BALB/c and outbred CD-1 mice (n = 5–6/group) were orally (intragastric) immunized with: (i) saline, (ii) dmLT alone or (iii) dmLT with U-Omp19 (150 µg). Three doses of dmLT were studied alone or plus U-Omp19: a dose of 25 µg used in clinical trials [22] or 12.5 µg and 2.5 µg of dmLT.

CD-1 mice were vaccinated at days 0, 28 and 42, whereas BALB/c mice were immunized following two protocols: (i) single dose or (ii) two doses (at days 0 and 28).

All mice were fasted 2 h before and 2 h after immunization.

### 2.5 Determination of antibody levels at feces and sera

Feces and sera were obtained weekly as described previously [32] to study LT- specific antibody responses (IgA and IgG in feces and IgG, IgG1, IgG2a and IgA in sera) by indirect ELISA. Hence, 96-well plates were coated with 0.1 µg/well of LT in carbonate buffer (15 mM Na_2_CO_3_, 35 mM NaHCO_3_, 0.2 g/liter NaN_3_, pH 8.6) overnight at 4 °C. Plates were washed with PBS-Tween 0.05% and blocked with 1% bovine serum albumin (BSA) for 1 h at 37 °C. Plates were then incubated with fecal extracts or sera (diluted in PBS containing 1% BSA). Incubations at 37 °C for 2 h for fecal samples and 1 h for sera samples were performed and then plates were washed and incubated with HRP conjugated anti-mouse IgA, IgG (SIGMA, St. Louis, MO, USA), IgG1 or IgG2a (Santa Cruz Biotechnology, Santa Cruz, CA, USA) for 1 h at 37 °C. Then, TMB (3,3́,5,5́tetramethylbenzidine) was added and reaction was stopped with H_2_SO_4_ 4 N and immediately read at 450 nm to collect end point ELISA data.

End-point cut-off values for serum titer determination were calculated as the mean specific optical density (OD) plus 3 standard deviation (SD) from sera of saline immunized mice and titers were established as the reciprocal of the last dilution yielding an OD higher than the cut-off. ELISA assay was performed the same day of feces collection.

Anti-U-Omp19 ELISA was performed as previously described [38].

### 2.6 Determination of antibody avidity

Anti-LT IgG avidity was measured in sera of immunized animals after last vaccination as previously described [39]. Briefly, 96-well plates were coated, washed, and blocked as described above. Then, sera samples were plated in duplicates for 1 h and plates were washed and incubated with either 6 M urea solution or PBS for 10 min at 37 °C. After washing, plates were incubated with anti-mouse IgG-HRP (SIGMA, St. Louis, MO, USA) and then washed and revealed with TMB. Reaction was stopped with H_2_SO_4_ 4 N and immediately read at 450 nm to collect end point ELISA data and calculate avidity indexes as the ratio of the O.D. in the urea-treated wells to untreated wells.

### 2.7 Patent mouse gut assay

After last immunization mice were challenged with LT following the patent (nonoccluded gut) mouse assay as previously described [40]. Briefly, the animals were fasted overnight and challenged orally with LT at doses of 75 µg to CD-1 mice and 50 µg to BALB/c mice in 0.5 ml saline solution. After 3 h of inoculation the animals were sacrificed, the entire intestine from duodenum to anus from each mouse was removed carefully to retain any accumulated fluid and residual mesentery was eliminated prior to weigh them. The carcass was weighed separately, and individual gut/carcass (G/C) ratio was calculated as indicator of intestinal fluid accumulation. The best dose of LT to challenge mice was chosen based on previous experiments administering different amounts of native LT to CD-1 or BALB/c unimmunized animals.

### 2.8 Neutralization of toxin-mediated intestinal fluid secretion

The neutralization capacity of sera was tested *in vivo* as previously described [23]. BALB/c mice were challenged intragastrically with 0.5 ml saline solution containing 50 μg of LT pre-incubated with dilutions of sera for 30 min at room temperature. After 3 h animals were sacrificed, the entire intestine from duodenum to rectum was carefully removed to retain any accumulated fluid, and the residual mesentery was removed prior to weigh. Entire intestine and large and small intestine were weighed. The carcass was weighed separately. LT-induced diarrhea is shown as gut/carcass ratio.

### 2.9 Data analysis

Statistical analysis and plotting were performed using GraphPad Prism 7 software (GraphPad, San Diego, CA). In the case of data from antibody levels (IgA, IgG, IgG1 and IgG2a), avidity in sera and feces samples and patent gut mouse assay data (with logarithmic transformation when necessary) were tested for normality and homoscedasticity before using parametric statistics (one-way ANOVA with Bonferroni post-test, two-way ANOVA or Unpaired T test). Normality was tested using the D'Agostino-Pearson normality test, and homogeneity of variances was tested using the Levene Median test. In cases where non-normality was suspected (data from *in vivo* LT neutralization experiments) statistical analysis was performed using non-parametric test (Unpaired Mann-Whitney T test). Results shown are representative of at least two independent experiments. The number of replicates per experimental group is five or six. Results were expressed as mean ± SEM. Significance level was set at p < 0.05.

## 3 Results

Oral co-delivery of U-Omp19 with dmLT increases anti-LT mucosal antibody responses and improves protection against heat-labile enterotoxin oral challenge in CD-1 mice.

Our first aim was to evaluate the effect of U-Omp19 co-delivery on dmLT immunogenicity in outbred mice. Though outbred animals may cause more variability in the experiments, they are more akin to the human population in terms of genetic diversity. We selected the outbred genetically heterogeneous CD-1 strain of mice because it has been used previously to investigate the protective capacity of different vaccine formulations against *Escherichia coli* intestinal colonization [13], [41]. Thus, CD-1 mice were orally (i.g) immunized at days 0, 28 and 42 with i) saline, ii) dmLT alone or iii) dmLT + U-Omp19. To investigate if U-Omp19 could have a dose sparing effect, three doses of dmLT (25, 12.5 and 2.5 µg) were evaluated alone or plus U-Omp19.

**Fig. 1:**
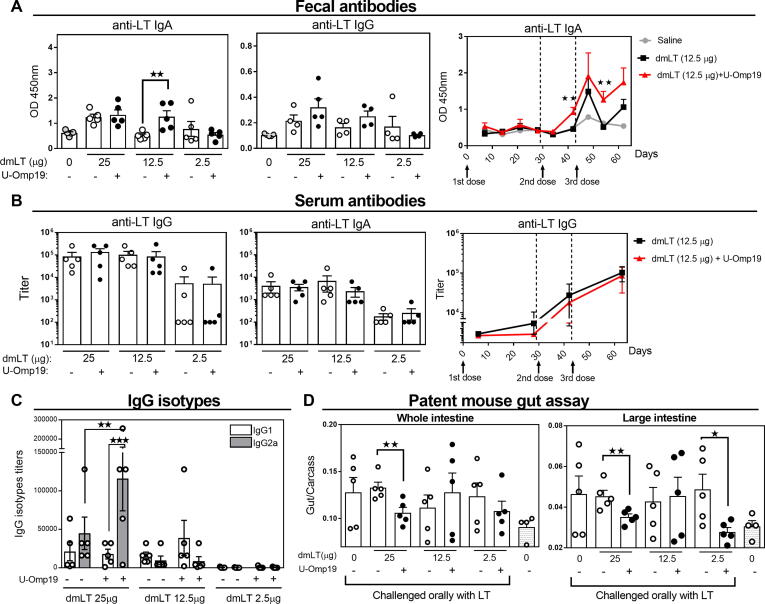
Oral co-administration of U-Omp19 with dmLT increases anti-LT Ab responses and protects against LT challenge in outbread mice. CD-1 mice were orally immunized at days 0, 28 and 42 with: (i) saline, (ii) dmLT (25, 12.5 and 2.5 µg/mouse) or (iii) dmLT + U-Omp19. Systemic and mucosal antibodies were evaluated by ELISA. A. anti-LT IgA and IgG levels in feces was evaluated two weeks after last immunization (left). Time course of anti-LT fecal IgA in the groups of mice immunized with dmLT (12.5 µg) alone or plus U-Omp19 (right). Results are shown as optical density (OD) 450 nm. **P < 0.01. One Way ANOVA with Bonferroni post-test. B. Titers of anti-LT IgG and IgA in serum three weeks after last immunization. C. IgG isotypes in serum of immunized animals. Anti-LT IgG1 and IgG2a titers were determined by ELISA three weeks after last immunization. **P < 0.01, ***P < 0.001. One Way ANOVA with Bonferroni post-test. D. Patent mouse gut assay. Immunized mice were orally challenged 1 month after last immunization with LT (75 μg/mice) and 3 h later the entire intestine from duodenum to anus was excised. Total intestine, large and small intestine sections and carcasses were separately weighed and individual gut/carcass weight ratios for each mouse calculated. *P < 0.05, **P < 0.01. Unpaired T test. Data points represent individual mice. Data from one representative experiment of two independent experiments.

Levels of anti-LT antibodies in feces and sera were evaluated by ELISA. Two weeks after second and third immunization, U-Omp19 co-delivery induced a significant increment in mucosal anti-LT IgA antibodies with 12.5 µg dose of dmLT in comparison with dmLT delivered alone. There was a slightly but not statistically significant increase in anti-LT IgG antibodies at feces in the group immunized with 25 and 12.5 µg of dmLT plus U-Omp19 in comparison with dmLT alone Fig. 1A. Time progression analysis of anti-LT IgA levels at feces from animals that were immunized with 12.5 µg of dmLT indicated that the antibody (Ab) response was low after first and second immunization and peaked after third immunization (Fig. 1**A)**. This response remained higher, but not statistically different to dmLT alone at three weeks after last immunization (Supplementary Fig. 1). In serum, there were no differences in the magnitude of anti-LT IgG or IgA titers between the groups with or without U-Omp19 at any of the doses of dmLT evaluated (Fig. 1**B**). Nevertheless, isotypes profile in sera changed after co-administration of U-Omp19 with dmLT where levels of IgG2a were significantly increased in comparison with the group that received the same dose of dmLT (25 µg) alone (Fig. 1**C**). Of note, anti-U-Omp19 antibodies were not detected in sera of any immunized group (Supplementary Fig. 2**A**).

**Fig. 2:**
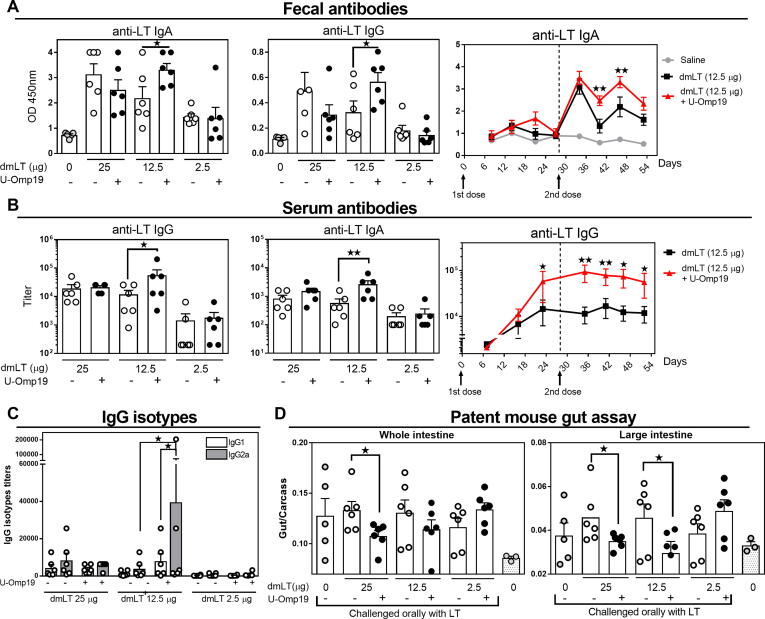
Oral co-administration of U-Omp19 with dmLT induces LT specific immune responses and protects against LT challenge in BALB/c mice. BALB/c mice were orally immunized at day 0 and 28 with (i) saline, (ii) dmLT (25, 12.5 or 2.5 µg/mouse) or iii) dmLT + U-Omp19. A. Anti-LT IgA and IgG levels were evaluated in feces by ELISA two weeks after second immunization (left). Time course progression of anti-LT IgA in feces in the groups immunized with dmLT (12.5 µg) alone or plus U-Omp19. Results are shown as optical density (OD) 450 nm. *P < 0.05, **P < 0.01. One Way ANOVA with Bonferroni post-test. B. Titers of specific anti-LT antibodies in serum two weeks after last immunization (left) and time course progression of serum anti-LT IgG in dmLT (12.5 µg) alone or plus U-Omp19 immunized groups (right). C. IgG isotypes in serum of immunized animals. Titers of IgG1 and IgG2a were determined by ELISA three weeks after last immunization. **P < 0.01, ***P < 0.001. One Way ANOVA with Bonferroni post-test. D. Patent mouse gut assay. Immunized mice were orally challenged with LT (50 μg/mice) and 3 h later the entire intestine from duodenum to anus was excised. Total intestine, large and small intestine sections and carcasses were separately weighed and individual gut/carcass weight ratios for each mouse calculated. *P < 0.05. Unpaired T test. Data points represent individual mice. Data from one representative experiment of two independent experiments.

Antibody responses are important to prevent ETEC bacterial adherence and toxins neutralization to avoid ETEC-associated diarrhea. Ability of vaccine formulation to prevent LT-induced diarrhea was evaluated by the patent mouse gut assay one month after last immunization. To do this, immunized CD-1 mice were challenged orally with LT (75 μg) and 3 h later mice were sacrificed, and each intestine and carcass was weighted. A significant protection was achieved in mice immunized with 25 μg dmLT plus U-Omp19 while dmLT alone did not protect against LT oral challenge (Fig. 1**D**). Protection was also observed with the dose of 2.5 μg dmLT plus U-Omp19 considering large intestine weight. These results indicate that U-Omp19 can increase dmLT immunogenicity and the efficacy to neutralize LT *in vivo* following an immunization protocol consisting of one primary immunization and two boosts in outbred CD-1 mice.

### 3.1 U-Omp19 in the oral vaccine formulation can help to reduce dmLT dose in BALB/c mice and improves protection against heat-labile enterotoxin oral challenge

To investigate if U-Omp19 improves dmLT immunogenicity in different genetic backgrounds despite their intrinsic variability we also tested BALB/c mice. We evaluated if U-Omp19 can help to reduce the number of doses administered, thus BALB/c mice were immunized two times with a first immunization at day 0 and a boost at day 28. Again, three doses of dmLT were evaluated (25 µg, 12.5 µg or 2.5 µg) with or without U-Omp19 in the oral formulation. Two weeks after second immunization there was an increment of anti-LT IgA and IgG in feces in the group of mice that received 12.5 µg of dmLT co-delivered with U-Omp19 (Fig. 2**A**), in comparison with dmLT immunization alone. Anti-LT IgA antibodies remained higher in the group immunized with dmLT 12.5 plus U-Omp19 up to three weeks after last immunization although the statistical differences in IgG antibodies were lost (Supplementary Fig. 3). Also, serum antibodies had shown a significant increment in anti-toxin IgG and IgA in the group that was immunized with 12.5 µg of dmLT plus U-Omp19 (Fig. 2**B**). After examining the progression of IgG titers at different time points, we observed that the increment in anti-LT IgG titers was maintained up to four weeks after the second immunization in dmLT 12.5 plus U-Omp19 group compared with the group administered with the same dose of dmLT alone (Fig. 2**B**). After evaluating IgG isotypes, we observed that IgG2a Abs predominate over IgG1 responses in the group of 12.5 of dmLT + U-Omp19. There were no differences in anti-LT Abs at feces nor at sera when using 25 or 2.5 µg of dmLT with or without U-Omp19 (Fig. 2**C**). Anti-U-Omp19 antibodies were not detected in sera of any immunized group (Supplementary Fig. 2**B**).

One month after last immunization mice were challenged orally with LT (50 μg) and patent mouse gut assay was performed. The highest dose of dmLT (25 μg) co-delivered with U-Omp19 induced a significant reduction in fluid secretion after LT challenge either after evaluating the whole intestine or large intestine weight (Fig. 2**D**). U-Omp19 co-delivery with the mid-dose of dmLT (12.5 μg) was also capable to reduce LT effect on the large intestine fluid secretion in immunized mice (Fig. 2**D**).

Thus, results obtained after the prime-boost protocol revealed that vaccine formulation containing U-Omp19 can help to induce systemic and mucosal Ag specific antibody responses in mice using a lower dose of dmLT (12.5 μg) while inducing protection after LT challenge.

**Fig. 3:**
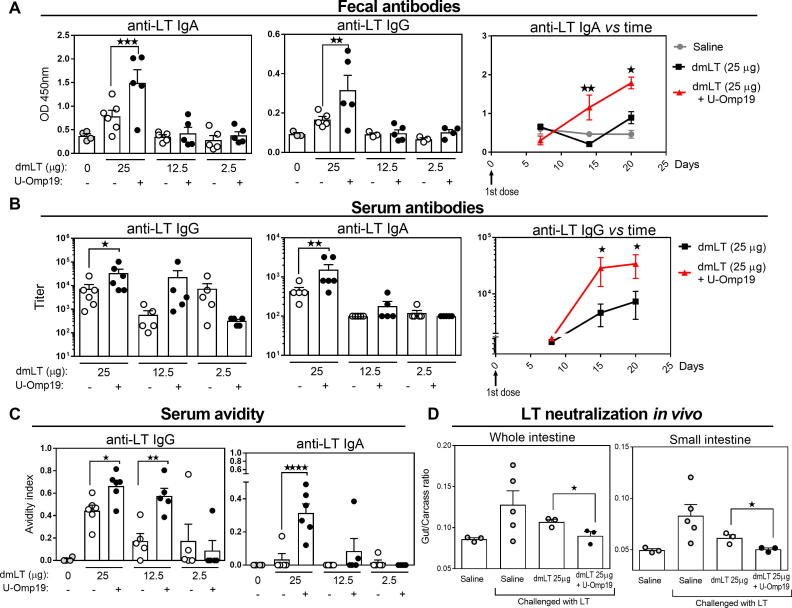
Single dose oral co-administration of U-Omp19 with dmLT increases anti-LT Ab responses that neutralizes LT induced diarrhea in BALB/c mice. BALB/c mice were orally immunized with (i) saline, (ii) dmLT (25, 12.5 or 2.5 µg) or (iii) dmLT + U-Omp19 at day 0. A. Anti-LT IgA and IgG were evaluated in feces by ELISA three weeks after immunization (left). Time course progression of anti-LT IgA levels of the groups immunized with dmLT (25 µg) alone and plus U-Omp19 (right). Results are shown as optical density (OD) 450 nm. **P < 0.01, ***P < 0.001. One Way ANOVA with Bonferroni post-test. B. Titers of specific anti-LT antibodies in serum three weeks after single immunization (left) and time course progression of anti-LT IgG of the groups immunized with dmLT (25 µg) alone or plus U-Omp19 (right). *P < 0.05, **P < 0.01. One Way ANOVA with Bonferroni post-test. C. Avidity of anti-LT IgG and IgA was evaluated in the serum of immunized animals three weeks after last immunization. Results are shown as Avidity Index (optical density (OD) after incubation with UREA/O.D. after incubation in PBS). *P < 0.05, **P < 0.01. One Way ANOVA with Bonferroni post-test. D. LT neutralization assay. LT was incubated with pooled sera from immunized mice during 1 h and then it was administered orally to mice. Three hours after challenge, animals were killed and the entire intestine from duodenum to anus was excised. Whole intestine, large and small intestine sections and carcasses were separately weighed and individual gut/carcass weight ratios for each mouse was calculated. *P < 0.05. Mann-Whitney T test. Data points represent individual mice. Data from one representative experiment of two independent experiments.

Finally, dose sparing strategy was evaluated after one immunization with the Ag dmLT. A single oral immunization protocol was performed in BALB/c mice testing again three doses of dmLT (25, 12.5 or 2.5 μg) alone or in presence of U-Omp19. Results showed that mucosal specific IgA and IgG antibodies were increased two weeks after single immunization when the highest dose of dmLT (25 μg) plus U-Omp19 was used, in comparison with dmLT delivered alone (Fig. 3**A**). This improvement was not observed when using the lower doses of dmLT (12.5 or 2.5 µg). Likewise, anti-LT IgG and IgA antibodies in serum were increased after oral immunization with the highest dose of dmLT plus U-Omp19 in comparison with dmLT delivered alone (Fig. 3**B**). Levels of systemic IgG isotypes were changed after co-administration of 25 μg dmLT with U-Omp19 in comparison with dmLT alone, in this case IgG1 titers were significantly increased three weeks after oral immunization (Supplementary Fig. 4). It is important to state that anti-U-Omp19 antibodies were not detected in sera of any immunized group (Supplementary Fig. 2**C**).

Since the ability of ETEC vaccines to induce mucosal antibodies with high avidity may also influence their protective efficacy we measured antibody avidity of IgG and IgA in serum from mice of all groups. There was a significant increase in anti-LT IgG and IgA avidity after a single oral administration of 25 or 12.5 µg of dmLT plus U-Omp19 in comparison with dmLT delivered alone (same dose) (Fig. 3**C**). Immunization protocols with one or two boosts did not modify antibody avidity in serum (data not shown).

Finally, *in vivo* neutralization of toxin mediated diarrhea was assessed by preincubating LT with a pool of sera from each group of vaccinated animals. Then, preincubated LT was used to challenge non-immunized animals and patent mouse gut assay was performed. Interestingly, sera from dmLT (25 µg) plus U-Omp19 vaccinated mice significantly inhibited LT effect on intestine inflammation compared with sera from the group immunized with dmLT alone (Fig. 3**D**).

These results together indicate that oral co-administration of U-Omp19 with dmLT can increase LT-specific mucosal and systemic antibody responses and improve avidity and neutralization capability of antibodies after a single dose immunization schedule in BALB/c mice. In summary, results demonstrated that U-Omp19 administration would be useful for antigen sparing strategies in oral vaccine formulations against ETEC.

## 4 Discussion

Usefulness of U-Omp19 as an oral vaccine adjuvant in mice has been previously reported. We have shown that protease inhibitor properties of U-Omp19 allow it to bypass the harsh environment of the gastrointestinal tract limiting co-administered Ag digestion and consequently increasing Ag amount at immune inductive sites. In addition, immunostimulatory properties of U-Omp19 induce mucosal and systemic Ag-specific immune responses (Th1, Th17 and CD8^+^ T cells) after oral co-administration with the Ag [33], [36], [37].

In this work, we evaluated the immunogenicity and protective efficacy of an oral formulation containing U-Omp19 and dmLT as antigen in mice. LT is one of the principal ETEC virulence factors and it has been studied as a potential vaccine antigen, as well as an adjuvant to induce mucosal immune responses [20]. The non-toxic LT double mutant (LTR192G/L211A or dmLT) has been shown to be immunogenic in animals and human trials and protective in animal models [28], [42]. Also, dmLT has demonstrated adjuvanticity in mice. Thus, this protein has the potential to be both a stand-alone vaccine as well as a mucosal adjuvant for other co-administered vaccine antigens [31]. Nevertheless, recent human clinical trials has demonstrated moderate immunogenicity at doses up to 50 µg of dmLT administered by oral or sublingual route [42]. Mucosal delivery of vaccines is more effective for eliciting mucosal immune responses, however, oral vaccination against enteric pathogens is difficult to achieve because of the gastric environment and the potential for inducing tolerance to the vaccine Ag . Our results demonstrated that co-administration of dmLT with U-Omp19 by oral route increased dmLT immunogenicity inducing specific mucosal and systemic antibodies leading to protection against LT enterotoxin challenge in mice. Indeed, it has been previously reported that protease inhibitor properties of U-Omp19 can increase the amount of CTB that reach mucosal surfaces after its oral delivery in mice enhancing its immunogenicity [37].

Different routes of administration studies have demonstrated clear differences in the doses of dmLT required to induce immune responses, standard dose of dmLT as adjuvant by oral route is 10 to 25 µg while for sublingual route is 1 to 5 µg [42]. Indeed, high doses of Ag can suppress the magnitude of responses and also bias to an antibody/Th2 response as it was seen in the clinical studies for ETVAX vaccine where 10 µg dmLT was superior to 25 µg [22]. In this work, we studied three different doses of dmLT and three different protocols of immunization. Higher antibody responses were observed after immunization with higher doses of dmLT (12.5 and 25 µg) in all immunization schedules. Following immunization protocols with one primary immunization and one or two boosts U-Omp19 increases anti-LT specific mucosal antibodies in the dose of 12.5 µg of dmLT while after a single oral dose only the highest dose of dmLT (25 µg) plus U-Omp19 could increase the levels of anti-LT antibodies in comparison with dmLT alone. Of note, U-Omp19 improved the induction of systemic anti-LT antibodies in the immunization protocols with fewer number of doses compared with the three-doses immunization protocol where there were no differences in the titers of Ag-specific IgG or IgA antibodies in presence or absence of U-Omp19. Thus, U-Omp19 seems to be crucial to increase mucosal antibodies when the Ag is given in low or suboptimal dose. In conclusion, addition of U-Omp19 to the vaccine formulation can improve anti-LT antibody responses with reduced number of administrations or Ag dose.

On the other hand, results of enterotoxin neutralization capacity of induced antibodies showed that animals immunized with the highest dose of dmLT plus U-Omp19 were capable to reduce intestinal fluid secretion after LT oral challenge in all the immunization schedules. The anti-toxin antibody responses observed were not totally consistent or correlated with the protective efficacy against LT challenge since the best dose of dmLT that induced significant mucosal antibody responses was not the same that prevented LT enterotoxicity after three doses of immunization. Although neutralizing antitoxin antibodies have been associated with ETEC protection [43] physiology of antibody immune responses could explain the discrepancy observed in our results. First, oral challenge with LT enterotoxin could trigger a local inflammatory response that stimulate antibody secreting B cells present in the inductive sites of the intestinal mucosa. In fact, the group of animals that was protected after LT oral challenge showed higher, but not significant, antitoxin mucosal antibodies after co-administration of U-Omp19 with 25 µg of dmLT (Fig. 1**A**). Moreover, the transport of systemic antibodies at the lumen of gastrointestinal tract via enterohepatic circulation should be taken in account (J. Clements, personal communication) [44]. In fact, the levels of Ab measured in the samples of feces or serum not always correlates with the amount of antibodies that can induce protection *in vivo*.

The capacity of an adjuvant to switch IgG isotypes is of significance for the outcome of humoral and cellular responses against the Ag. IgG2 isotype has the highest affinity for the Fcγ receptors that mediate antibody-dependent cellular cytotoxicity and phagocytosis (ADCC and ADCP) [45]. Therefore, adjuvants such as the TLR4 agonist SLA-SE that promote IgG2a class-switching in mice may be beneficial to block either adherence via colonization factors or cAMP flux caused by LT and/or ST [46]. U-Omp19 promoted the production of anti-LT IgG2a antibodies over IgG1 in serum of immunized animals after two or three immunizations (Figs. 1**C and** 2**C**). On the contrary, after a single oral dose dmLT plus U-Omp19 induced higher levels of IgG1 antitoxin antibodies. It has been proposed that the ability of ETEC vaccines to induce antibodies with high avidity may also influence their protective efficacy [13], [18]. Of note, the inclusion of U-Omp19 in dmLT vaccine formulation also increased the avidity against LT of IgG elicited Ab in comparison with the administration of dmLT alone (Suppl. Fig. 4 **and** Fig. 3**C**). Indeed, antibody avidity has been shown to correlate with the presence of Ag-specific memory B cells following enteric pathogen infection [39].

Vaccine manufacturing process is time-consuming and many times the production capacity is limited [7]. Therefore, it is important to improve vaccine manufacturing strategies and reduce its cost by developing new techniques or reducing antigen dose. Incorporation of Ag-sparing adjuvant(s) into vaccine formulations cans solve this problem and could be critical in the case of pandemic outbreaks [47], [48]. Admixing U-Omp19 adjuvant with a vaccine could be an effective and efficient approach for antigen sparing, and consequently improvement of vaccine production capacity. On the other hand, inclusion of the LT toxoid component in the vaccine may help broaden protection to potentially other enteric pathogens like Salmonella or Campylobacter [25], [49]. Therefore anti-LT based vaccine can be protective against a broader array of ETEC pathotypes [50]. Interestingly, several studies now indicate that dmLT can also provide nonspecific protection from disease and that may be due to activation of innate immunity [20], [25]. Thus, it will be interesting to study in future works if U-Omp19 modulate or increase innate immunity induced by dmLT.

Overall, an oral vaccine formulation containing dmLT and U-Omp19 can induce antitoxin antibody responses and can be effective against ETEC induced diarrhea. This work served as proof of concept on the inclusion of dmLT together with U-Omp19 in a vaccine formulation against ETEC. Thus, our next steps are combining dmLT, U-Omp19 and new ETEC Ags in oral vaccine formulations.

## Declaration of Competing Interest

The authors declare that they have no known competing financial interests or personal relationships that could have appeared to influence the work reported in this paper.
