# Supplementary material for: U-Omp19 from Brucella abortus increases dmLT immunogenicity and improves protection against Escherichia coli heat-labile toxin (LT) oral challenge
Source: Vaccine. 2020 Jul 6;38(32):5027–35. doi: 10.1016/j.vaccine.2020.05.039 (PMC7327514; doi:10.1016/j.vaccine.2020.05.039)
Supplement: Supplementary data 1 [file mmc1.docx]

**Supplementary Figure 1**


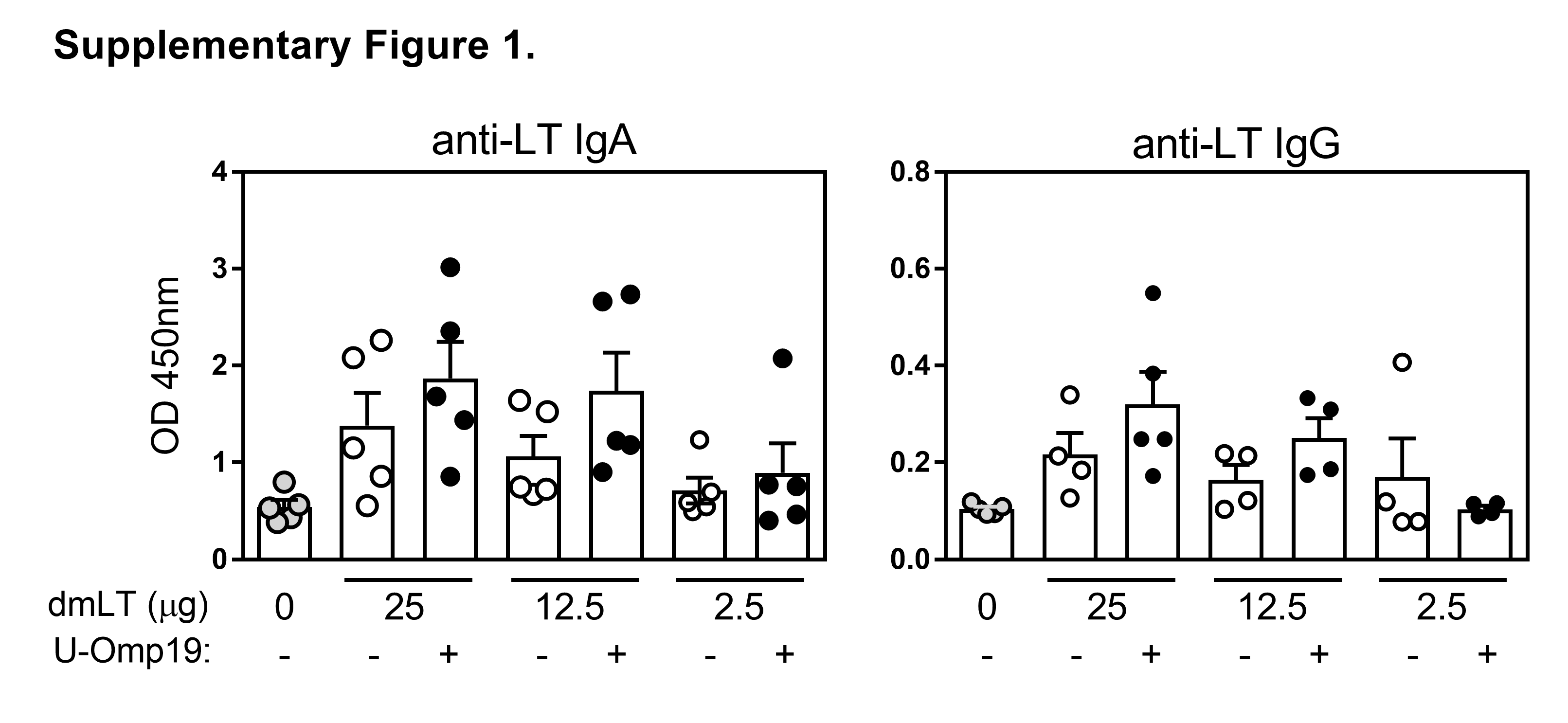


**Supplementary Figure 1.** CD-1 mice were orally immunized with i) saline, ii) dmLT (25, 12.5 or 2.5 µg) or iii) dmLT+U-Omp19 at day 0, 28 and 42. Levels of anti-LT IgA and IgG in serum were determined by ELISA three weeks after last immunization. Data points represent individual mice Data from one representative of two independent experiments.


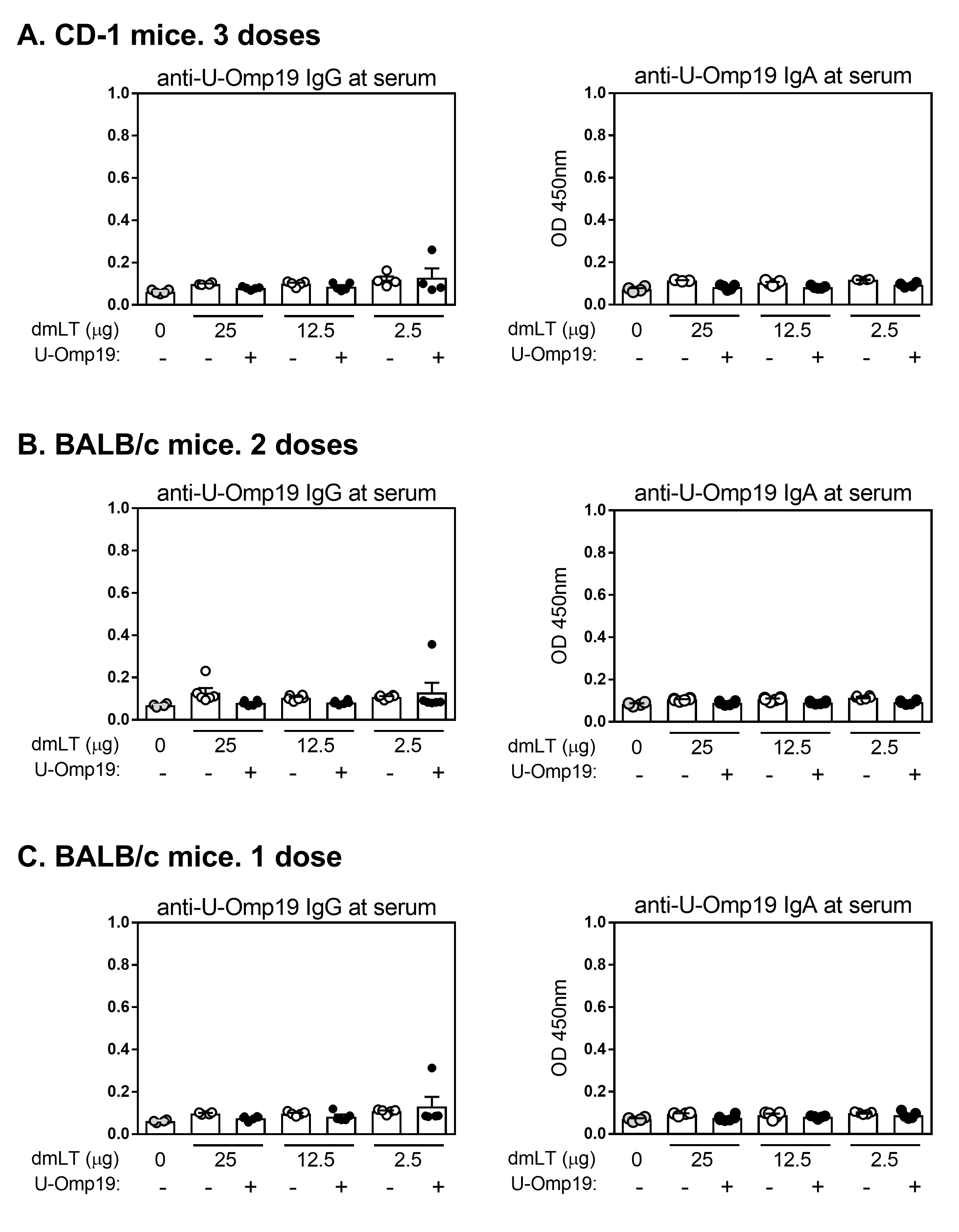


**Supplementary Figure 2**

**Supplementary Figure 2. A.** CD-1 mice were orally immunized with i) saline, ii) dmLT (25, 12.5 or 2.5 µg) or iii) dmLT+U-Omp19 at day 0, 28 and 42. Levels of anti-U-Omp19 IgA and IgG in serum were determined by ELISA three weeks after last immunization. **B**. BALB/c mice were orally immunized with i) saline, ii) dmLT (25, 12.5 or 2.5 µg) or iii) dmLT+U-Omp19 at day 0, and 28. Levels of anti-U-Omp19 IgA and IgG in serum were determined by ELISA three weeks after last immunization. **C**. BALB/c mice were orally immunized with i) saline, ii) dmLT (25, 12.5 or 2.5 µg) or iii) dmLT+U-Omp19 at day 0. Levels of anti- U-Omp19 IgA and IgG in serum were determined by ELISA three weeks after last immunization. Data points represent individual mice. Data from one representative of two independent experiments.

**Supplemental Figure 3**

**Supplementary Figure 3.** BALB/c mice were orally immunized with i) saline, ii) dmLT (25, 12.5 or 2.5 µg) or iii) dmLT+U-Omp19 at day 0, and 28. Levels of anti-LT IgA and IgG in serum were determined by ELISA three weeks after last immunization. Data points represent individual mice. Data from one representative of two independent experiments.


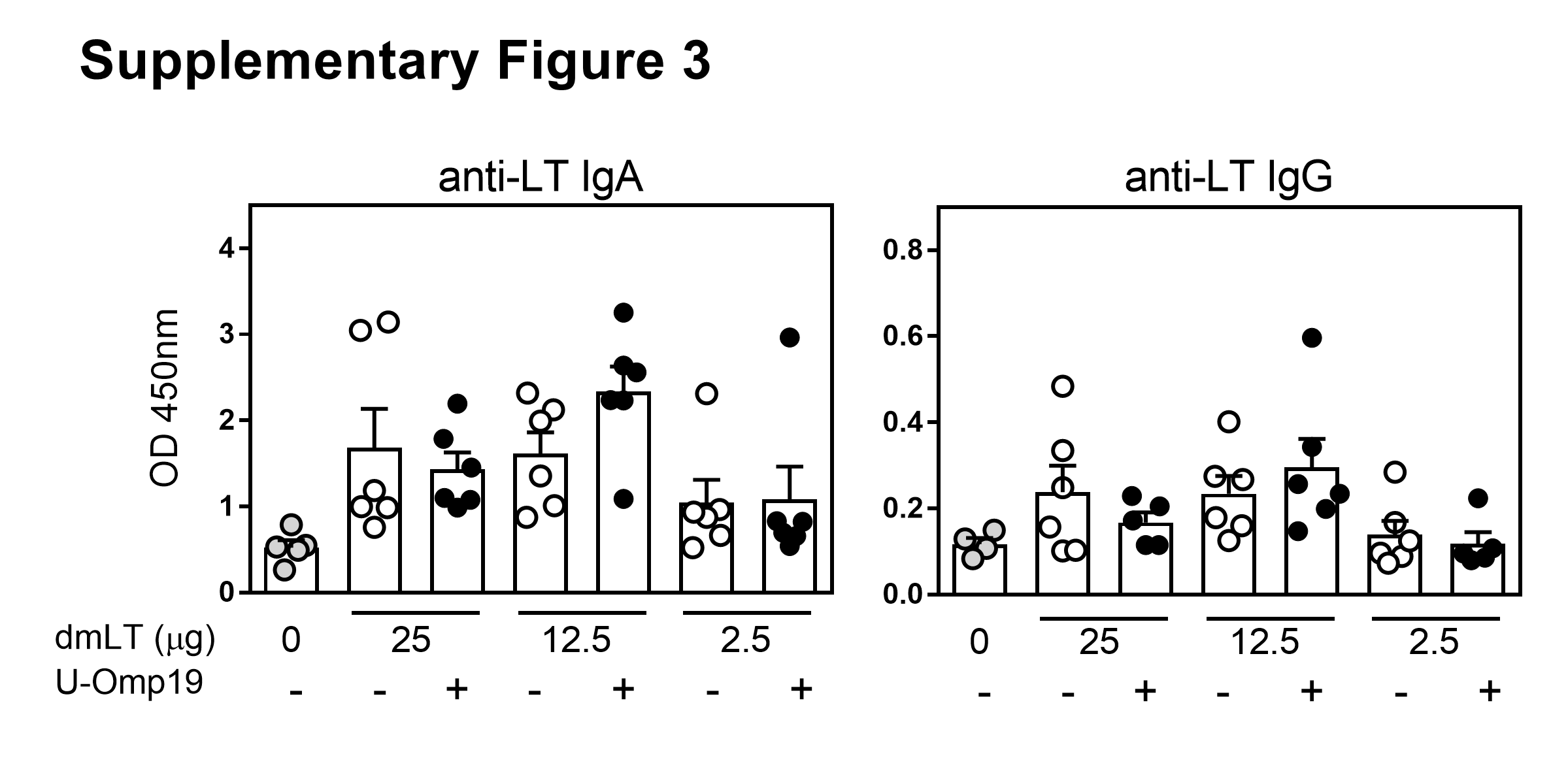


**Supplementary Figure 4**

**Supplementary Figure 4.** BALB/c mice were orally immunized with i) saline, ii) dmLT (25, 12.5 or 2.5 µg) or iii) dmLT+U-Omp19 at day 0. Titers of anti-LT IgG1 and IgG2a were determined by ELISA three weeks after a single oral dose. **P<0.01, ***P<0.001. One Way ANOVA with Bonferroni post-test. Data points represent individual mice. Data from one representative of two independent experiments.
